# Policy guidance on threats to legislative interventions in public health: a realist synthesis

**DOI:** 10.1186/1471-2458-11-222

**Published:** 2011-04-10

**Authors:** Geoff Wong, Ray Pawson, Lesley Owen

**Affiliations:** 1 NIHR Clinical Lecturer, Research Department of Primary Care and Population Health, UCL, Upper Third Floor, Royal Free Hospital, Rowland Hill Street, London, NW3 2PF, UK; 2 Professor of Social Research Methodology, School of Sociology and Social Policy, University of Leeds, Leeds, LS2 9JT, UK; 3 Senior Analyst, National Institute for Health and Clinical Excellence (NICE), MidCity Place, 71 High Holborn, London, WC1V 6NA, UK

## Abstract

**Background:**

Legislation is one of the most powerful weapons for improving population health and is often used by policy and decision makers. Little research exists to guide them as to whether legislation is feasible and/or will succeed. We aimed to produce a coherent and transferable evidence based framework of threats to legislative interventions to assist the decision making process and to test this through the 'case study' of legislation to ban smoking in cars carrying children.

**Methods:**

We conceptualised legislative interventions as a complex social interventions and so used the realist synthesis method to systematically review the literature for evidence. 99 articles were found through searches on five electronic databases (MEDLINE, HMIC, EMBASE, PsychINFO, Social Policy and Practice) and iterative purposive searching. Our initial searches sought any studies that contained information on smoking in vehicles carrying children. Throughout the review we continued where needed to search for additional studies of any type that would conceptually contribute to helping build and/or test our framework.

**Results:**

Our framework identified a series of transferable threats to public health legislation. When applied to smoking bans in vehicles; problem misidentification; public support; opposition; and enforcement issues were particularly prominent threats. Our framework enabled us to understand and explain the nature of each threat and to infer the most likely outcome if such legislation were to be proposed in a jurisdiction where no such ban existed.

Specifically, the micro-environment of a vehicle can contain highly hazardous levels of second hand smoke. Public support for such legislation is high amongst smokers and non-smokers and their underlying motivations were very similar - wanting to practice the Millian principle of protecting children from harm. Evidence indicated that the tobacco industry was not likely to oppose legislation and arguments that such a law would be 'unenforceable' were unfounded.

**Conclusion:**

It is possible to develop a coherent and transferable evidence based framework of the ideas and assumptions behind the threats to legislative intervention that may assist policy and decision makers to analyse and judge if legislation is feasible and/or likely to succeed.

## Background

Legislation is often considered by policy and decision makers as one of the most powerful weapons for improving the health of populations[1]. Numerous examples exist internationally and legislation either seeks to prescribe (e.g. the wearing seat belts in vehicles) or proscribe (e.g. banning smoking in public place) behaviours that impact on health. Legislation can improve the health of populations (as exemplified by tobacco control laws [2,3]), but it can also fail spectacularly [4] and have unintended outcomes[5].

When legislation is being considered, it is not always immediately apparent that it is likely to face a tortuous and long journey from conception to enactment and enforcement[6]. Multiple 'threats' (e.g. poor drafting, public opinion and lobby group opposition) may well impede its success[7].

Researchers have studied the 'on the ground' implementation of laws and/or their population health impacts. But little research has tried to draw together the evidence to produce a framework to help policy and decision makers on the suitability of opting for legislation in the first place and/or the likelihood that it will 'work'. Policy and decisions makers do frequently want their 'breaking ideas' tested and it is possible to review interventions that are in their infancy using realist synthesis.

Realist synthesis is a theory-driven and interpretive type of systematic review method. It is based on a realist philosophy of science which argues that we can improve our understandings of reality because "the real world" constrains the interpretations that we can reasonably make of it. Realism can be used to help us understand the social world. When used in this way it acknowledges the existence of an external social reality and the influence of that reality on human behaviour. Its goal is more to provide plausible explanations (for example to 'why?', 'how?' questions) than summative judgments about interventions[8]. We have used the realist synthesis method to help us construct our framework as it has enabled us to look inside the 'black-box' of what might happen when public health legislation is planned and implemented. More specifically, we have conceptualised legislative interventions as complex social interventions - where outcomes arise in a non-linear way thought multiple human interactions and decisions under the influence of a myriad of contextual factors. Such interventions present a formidable challenge to reviewers [9] and our hope is that our realist synthesis review has been able to deepen our understanding of the threats involved[8].

## Aim

In this realist synthesis we aimed to develop a coherent and transferable framework of the threats behind a legislative intervention that may assist policy and decision makers to analyse and judge if a legislative option is feasible and likely to succeed. Our framework of threats to the programme theory of public health legislation will highlight the main challenges that a piece of legislation aimed at improving population health might face. It is derived from an overview of public health legislation and tested and refined from evidence drawn from the 'case example' of banning smoking in private vehicles carrying children.

## Methods

**Figure 1:**
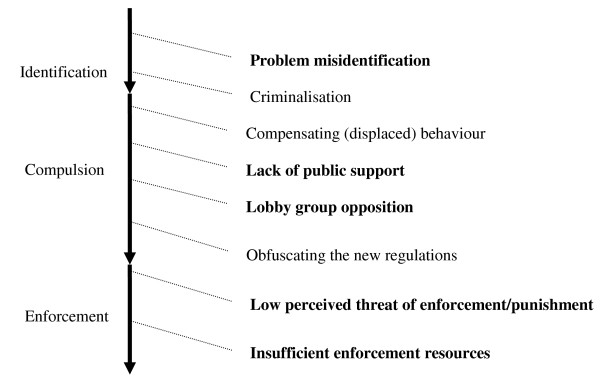
**Simplified diagrammatic representation of potential threats to legislation (our initial framework)**. Threats in bold font are the ones which were most prominent and relevant in our test case study of legislation banning smoking in vehicles carrying children. This figure has been highly simplified and for illustrative purposes has displayed the potential threats in a linear sequence.

The review team (RP, GW and LO) had been funded to undertake a review of the use of legislation in public health in partnership with a policy making body (NICE). In our initial discussions on the focus (or 'cut') of our review, we became aware that legislation was often proposed as an option by policy and decisions making bodies to improve public health. However little guidance was available in early policy decision making on the feasibility and/or the likelihood of success of such a move. We therefore agreed that an important area to focus on and address was guidance for policy and decision makers on the 'threats' proposed legislation might face from conception to the statute books and enforcement. To develop our framework on the threats to the programme theory of public health legislation we started out by conducting a rapid review of broad areas of public health legislation (covering everything from gun amnesties to food labelling) trying to uncover what had been the sticking points in legislation and how (if at all) they had been circumvented. This outline review lead to the construction of a provisional framework for reviewing the family of legislative interventions (as described in Figure 1). Further details of this initial overview has been described elsewhere[7]. Beginning with this framework and through discussions (and with reference to other interested stakeholders) we focused on a subset of themes that seemed most relevant in respect to the intervention in question. In our case we deliberately sought input from the NICE officer seconded to our project. Interested parties can include 'sceptics' and so we also selected themes based on issues that were proving politically contentious in debate surrounding legislation that sought to ban smoking in vehicles carrying children (for more details on why we selected this topic area see below). This influence is described in the paper: *The Today Programme's Contribution to Evidence-based Policy*[10]. On the basis of these considerations we narrowed our review further to the issues raised in Table 1.

**Table 1:** The key questions that need to be addressed in the identified threats to legislation banning smoking in vehicles carrying children

1. PROBLEM MISIDENTIFICATION
Is the severity of the problem sufficient to justify a law?
a. Is it possible to show that exposure to second-hand smoke in cars leads to ill-health?
b. What toxicity levels are encountered in a car when cigarettes are smoked?
c. Does ventilation make a difference?
d. Are the toxicity levels comparable to other risky environments in which smoking bans already operate?
e. How does the potential harm compare to formally approved air quality standards?
2. LACK OF PUBLIC SUPPORT
**Is there likely to be public support for such a law?**
a. What is the overall magnitude of support for such a law?
b. What are the levels of support amongst smokers?
c. What is the motivation behind public support?
d. Does endorsement depend on the extent and success of previous smoking bans in work and public places?

3. LOBBY GROUP OPPOSITION
**Is there likely to be effective pressure group opposition?**
a. Has the Tobacco lobby opposed this particular ban?
b. Are they likely to do so in future?

4. ENFORCEMENT
**Is the law enforceable?**
a. Given that the potential infraction is fleeting and localised will smokers fail to comply assuming there is little risk of being caught?
b. Given limited resources, the difficulties of detection and the fact that the law addresses a public health issue will the police act significantly on enforcement?
c. What other measure need to be incorporated to encourage compliance and enhance enforcement?

We wanted to test our framework on a topic area that would be relevant to our policy making partner. The duration (one year) and resources we had at our disposal meant that pragmatically we were only able to test our framework against one 'case' example. After discussions we agreed to test our framework on the threats that lay behind legislation that banned smoking in vehicles carrying children as this was being (at the time we started our project) advocated in the United Kingdom (and other countries) [10,11], viewed by many as being 'controversial' [12] and as yet unevaluated[13]. As this legislation remains formally unevaluated, it provided a most challenging example (especially as it involves private/personal space) through which to test and our framework - in other words allowing us to make inferences about what the passage of such legislation might be if policy and decision makers were to propose it. Our decisions above had implications on the degree to which we could 'fully' test the utility of our framework and we discuss this issue further later on.

Our searching process did not aim to be exhaustive but to seek out a representative body of literature on which to test and refine theory[14]. Initially, we developed, piloted and ran searches in five electronic data bases looking for any article types that included; children, smoking and vehicles (using relevant Boolean operators, wildcards, controlled terms and free text). We searched through the reference lists of the included articles (pearling) to identify further studies. No date limits were applied and articles in any language were eligible. Searching was an iterative process such that we performed additional purposive literature searches whenever additional data was required for theory testing.

As we were unable to find any formal studies that evaluated the enforcement of smoking bans in vehicles carrying children, we deliberately chose to seek out studies which examined the closely related topics of the enforcement of cellular phone use and child restraints in vehicles. Our logic for searching in these areas were that they involved enforcement of 'in vehicle' behaviours that were potentially equally hard to enforce and (in the case of child restraints) involved the safeguarding of children. As this was a sub-component of our framework we only searched MEDLINE and used pearling to identify additional studies. When additional data was required for testing of other sub-components of our framework we developed, piloted and ran additional searches only in MEDLINE but included pearling. The searching and screening of citations for inclusion was undertaken by GW and was in two phases - firstly of title, abstract and keywords (where available) and secondly of the full text of potentially relevant articles (from the first screen). Articles were included based on our inclusion criteria for each search undertaken (for example for our initial search it needed to contain information on smoking, children and vehicles). Included articles were not judged on any specific measure of their overall quality but on their ability to provide data for theory testing (i.e. their relevance)[8].

We extracted the information about the included studies' characteristics into an Excel spreadsheet. The data extracted included; article details; country of origin; article type; aim of article; research method (if applicable); and a brief summary of how the article informed the review. The main function of our spreadsheet was to provide an easy to access overview of all relevant and included articles. Where technically possible an electronic version (e.g. pdf) of included studies were imported into NVivo 8 (http://www.qsrinternational.com/ - NVivo 8 file available at request from authors) and verbatim sections of text were coded against codes (nodes in NVivo terminology) derived from our initial programme theory to enable transparency (and easy of retrieval) in our theory testing process. We created additional codes as our synthesis progressed in order to capture new theory testing data and we deliberately revisited articles that we had coded earlier in order to ensure completeness and consistency in coding[14].

Data synthesis was undertaken either by RP and/or GW and synthesis results were regularly shared and discussed within the review team to ensure validity and consistency in the inferences made. Specifically (where relevant) we attempted to identify prominent recurrent patterns of contexts and outcomes (demi-regularities) in the data and then sought to explain these through the means (mechanisms) by which they occurred. For example, we noted that in our included articles self reported public support for a ban on smoking in vehicles carrying children was often found to be high amongst smokers. During data synthesis we would then aim to provide an explanation of this demi-regularity through the identification mechanism(s). As we delved further into our included articles and beyond (through our aforementioned purposive searching) for an explanation, data emerged that smokers harboured within them the wish to want to protect children from harm and also regret at having started smoking. We interpreted these as (realist) mechanisms and for the former was able to find substantive (middle-range) theory in the form of the Millean principle [15] to explain its interaction with context to influence outcomes. When additional studies were sought to enable programme theory testing, data handling processes (as described above) were repeated.

## Results

### Framework structure

Our initial framework consisted of eight separate threats that a proposed piece of legislation might have to face from conception to enforcement. These threats include: problem misidentification, criminalisation, compensating behaviour, lack of public support, lobby group opposition, obfuscating the new regulations, low perceived threat of enforcement and insufficient enforcement resources (see Figure 1)[7]. When we tested our initial framework on our case study (banning smoking in vehicles carrying children), not all threats were either relevant and/or reported. We found little data on the passage of such a law being hampered by the criminalisation (of offending parents), compensatory behaviour of smokers and obfuscation of the legislation. This does not mean that these threats are not important in other legislative arenas, but more that we were unable to find the necessary data to inform us of their relevance when applied to our specific case study. Hence our results only focus on the four threats that we were able to find data and relevance for in our included studies, namely - problem misidentification, lack of public support, lobby group opposition and enforcement (a combined threat that includes both low perceived threat of enforcement/punishment and insufficient enforcement resources).

### Search and study characteristics used in synthesis

**Figure 2:**
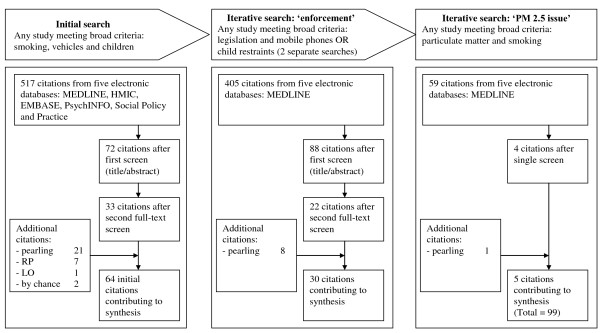
**Flow diagram illustrating search process and article disposition**.

Our iterative searches provided us with a total of 99 studies with which to test our framework (Figure 2). The characteristics of each of the included studies and also the 'threat' they contributed data to for theory testing and refinement is provided in Additional file 1, Table S1.

In brief summary of our 99 included studies all were in English, they dated from 1987 to 2009 and were all from developed nations. 74 (74.7%) reported primary research, 17 (17.2%) were reviews (of various types), 4 (4%) letters, 3 (3%) discussion pieces, 2 (2%) reports, 1 (1%) (a news article) and 1 (1%) a fact sheet (some articles reported on more than one item). Within primary research, the single most commonly used method was the 'one off' questionnaire survey (35(35%)). There were 17 (17%) quasi-experimental studies (9 (9%) used direct observation only, 5 (5%) databases and 3 (3%) observation and questionnaires). 12 (12%) were experimental studies (all examining air quality), 3 (3%) qualitative studies, 2 (2%) whole programme evaluations and 7 (7%) used other methods.

### Framework testing

In this section, 'threat' by 'threat', we report in more detail the results of how and to what extent each threat was manifest in order to pinpoint where such a law might prevail and where it might falter.

We found that our initial framework correctly identified the reported threats to legislation banning smoking in vehicles carrying children. We combined the last two threats into 'enforcement' as there was much overlap from our data. Further refinement of our framework was needed to increase its relevance specifically to our case study example. Thus within each of the threats, we identified additional key question (highlighted in bold in Table 1) for each of the legislative threats.

#### Problem misidentification - Is the severity of the problem sufficient to justify a law?

To be effective, legislation needs to target the appropriate 'problematic' issue. In our case study of smoking bans in vehicles carrying children, the assumption is that second hand smoke (SHS) is the problem as opposed to (for example) driver distraction whilst smoking. Within our included studies, we identified that SHS was almost always identified as being the problem. We thus worked with this assumption and identified an additional issue regarding the toxicity of SHS. Whilst there is no shortage of evidence indicating that SHS can in general be harmful [16], can we uncritically assume that SHS exposure in the microenvironment of a car is as harmful when compared to other environments? Associations of ill health to SHS exposure in vehicles have been demonstrated (as exemplified by Evans and Chen)[17]. Evidence indicates that in vehicle toxicity levels can be high[18-23]. Air quality studies under different conditions have been undertaken (see Table S1) and these clearly indicate that peak toxicity levels (measured in PM2.5 - particulate matters of 2.5 microns diameter) can increase by up to a 1000 fold from ambient levels[22,23]. We specifically explored the effects of ventilation on PM2.5 levels as this 'solution' was often put forward by opponents to such a ban. Experimental studies of the effects of ventilation on in-car air quality indicate that whilst ventilation can lower PM2.5 levels substantially, they remained above ambient levels[20]. When in vehicle PM2.5 levels are compared to other smoking environments (e.g. bars [24-26]) and also formally approved air quality standards [27], then it becomes even clearer that in vehicle levels can be comparable to other smoking environments and are by large orders of magnitude above approved air quality standards.

Our findings for this threat indicate that policy and decision makers would be right to assume that SHS does pose a sufficient problem to justify legislative action. SHS levels may in certain circumstances produce toxicity levels that are many orders of magnitude higher than accepted 'clean air' standards and be comparable to other situations where bans already operate (e.g. in pubs). Our findings here indicate the value of identifying the risks with greater precision especially as such information may be used to counter any proposed 'solutions' to harm (in this case ventilation).

#### Lack of public support - Is there likely to be public support for such a law?

Public support underpins the acceptability of a law and is perhaps best summed up by the words of suffragette Carrie Chapman Catt, '*No written law has ever been more binding than unwritten custom supported by popular opinion'*.

Multiple questionnaire surveys from different richer democratic nations (almost all with varying degrees of tobacco control measures in place) and at various points in time have addressed the question of the magnitude of support for such a ban. The surveys report levels of support ranging from 46 to 90.3% (mean 70.8%) for non-smoker [28,29] and 23.2 to 95.9% (mean 59.3%) for smokers [28,30] (see Table 2 for more details). There is a high magnitude of support for such a ban even amongst smokers. The data from our included studies indicates that the mechanism behind their motivation for such levels of support appears to be the overwhelming wish amongst both non-smokers and smokers to wish to protect children from harm. In effect responders were voicing their support for the Millian principle of their willingness to sacrifice personal liberties in the interest of protecting the 'vulnerable' from harm[15]. In addition for smokers, we found evidence that (the mechanism of) regret may have had an important influence[31]. Smokers appear to have a 'near-universal experience of regret' of belonging to their reference group (of being smokers) and so seemed much less willing to recruit others (especially children) to their ranks.

**Table 2:** Levels of self declared support for and/or practice of smoking bans in vehicles carrying children reported in included studies by smoking status (in date order)

Study	Year of survey data collection	Type of data reported (support and/or practice of ban)	Country	Cars		
				
				Non-smokers and smokers support/practice %	Non-smokers support/practice %	Smokers support/practice %
Bauman et al[46].	1994	Support	Australia, NSW	72		63

Norman et al[47].	1996/7	Practice	USA, California	66.0 (16.0 *)		

Walsh et al[48].	2000	Support and practice [in brackets]	Australia, NSW		58.8 [86.7]	44.7 [39.8]

Kegler et al[49].	2000	Practice	USA, Oklahoma State (North East)		67.4	12.8

Binns et al[50].	2001	Practice	USA, Chicago		83.0	58.0

King et al[51].	2001	Practice	USA, nationwide (African Americans only)		84.1	21.4

McMillen et al[52].	2002	Practice	USA, nationwide	83.2 (urban) 68.7 (rural) **		

Gonzales et al[53].	2003/4	Practice	USA, Albuquerque (Hispanics only)	81.0		

Walsh et al[54].	2004	Support	Australia, NSW		55.6	50.5

Leatherdale et al[55].	2004	Support	Canada, nationwide		91.8	72.9

Kegler et al[56].	2004/5	Practice	USA, Georgia (South West)	36.8 (40.4 *)		

Leatherdale et al[29].	2006	Support	Canada, nationwide		90.3	79.2

Jalleh et al[57].	2006#	Support	Australia, Western Australia		87	80

Thomson et al[30].	2007/8	Support	New Zealand, nationwide			95.9

Dunn et al[58].	2008	Support	Australia, Queensland		82.9***	76.9***

Key: * % support if ban was only partial

** % support differentiated by rural and urban responders

# year of publication

*** % support refers only to children aged 12 or under present in the car

There was less clear evidence on whether the pre-existence of other smoke free legislation facilitated the introduction of vehicle smoking bans, often referred to in our included studies as the 'denormalisation' theory[32]. Smokers reported being aware that it was becoming more and more unacceptable to smoke but it was not clear what effect this had on their level of support for such a ban. We did however find clear evidence that pre-existing legislation that encroached on personal freedoms in vehicles did go some way to diffusing the debates around the 'private space' argument.

In the data from our included studies (which are derived from the context of richer democratic countries with existing but varying degrees of tobacco control) self-reported support for a smoking ban in vehicles carrying children was high amongst both non-smokers and smokers. We were able to identify two important mechanisms (the wish to protect children and regret) which appeared to explain much of this level of support. Whilst it is likely that within any jurisdiction 'local' support for such a ban would predictably vary, we can infer that if a population supported the Millian principle and/or (if smokers) experienced regret, then support would likely be high.

#### Lobby group opposition - Is there likely to be effective pressure group opposition?

All legislation is likely to face some degree of opposition from parties with vested interests[1]. The literature, especially in tobacco control is replete with clear examples of opposition to further tobacco control measures. However, can we assume that all tobacco control measures face the same ferocity of opposition?

Limited evidence indicates that when such legislation was under consideration in Australia, no opposition was mounted by the tobacco industry[6]. To make any further inferences, we had to undertake more purposive searching looking specifically for publically available statements from the tobacco industry on smoking with relation to children, past attempts by the tobacco industry to lobby and opposed tobacco control measures and also the outcome of such efforts. The aim of such additional searching was to construct an overview of what limitations there might be on tobacco companies specifically in relation to smoking bans in vehicles carrying children.

We found that the vast majority of tobacco companies wished publicly to be seen as socially responsible and thus not support smoking in children (see for example http://www.jti.com/cr_home/cr_positions/cr_positions_youth_smoking). In addition in 1998 the major tobacco companies signed the US Master Settlement Agreement of 1998 which prohibits any activity targeting tobacco products at youths[33]. Our included studies indicate also that any opposition from the tobacco industry itself has been be readily and effectively countered by the smoke free lobby through the Millian principle[6,34,35]. Put together, we can infer that specifically for a smoking ban in vehicles carrying children, lobbying and opposition may be muted. Three important contextual constraints are apparent that (if present) limit and may be used to counter opposition and lobbying, namely - the importance a society places on enacting Millian principles; the extent to which the US Master Settlement Agreement of 1998 is legally enforced; and the extent to which tobacco companies enforce (or are forced to enforce) their own public statements on smoking and children.

Having an understanding of contextual influences on how opposition might respond against a piece of legislation enables inferences to be made about how likely it is to occur. This may well also allow countering moves to be prepared. An appreciation of the opposition's 'room for manoeuvre' appears to be of value in further refining possible inferences.

#### Enforcement - Is the law enforceable?

A frequently cited criticism of a smoking ban in vehicles carrying children is that it is not only un-enforceable but also not the role of the police to enforce public health legislation[36]. However, are these assumptions supported by data?

Our analysis of the data from the enforcement of bans on mobile phone usage and requirement for child restraints in vehicles indicates that the presence of legislation on its own is not enough to ensure compliance. We noted that when legislation was enacted, compliance either did not change or (where data was available) it increased but levels return to (almost) pre-legislative levels within months suggesting an inverse 'U'-shaped curve - see Tables 3 (mobile phones usage) and 4 (child restraints usage) for more details. Active enforcement strategies (e.g. 'crack-downs', publicity, warnings) that are geared towards increasing the (mechanism of the) perception of 'being caught' are able to sustain compliance for longer[37,38]. The impact of such measures is well recognised in the literature [39] and they appear to act by increasing perceptions of the likelihood of being 'caught'[40]. The child restraints in vehicles data adds a further dimension as they reported that the motivation for compliance is not the fear of the penalty, but the wish to protect children[41].

**Table 3:** Impact of legislation on non-compliance reported by included studies on mobile phone usage in vehicles

		Data for non-compliance			
			
Study	Location	Pre legislation % (sampling interval before legislation)	Post legislation % - Time 1 (sampling interval after legislation)	Post legislation % - Time 2 (sampling interval after legislation)	Method used to obtain data
Johal et al[59]. and Hussain et al[60].	UK, Birmingham	1.85 (10 weeks)	0.97 (10 weeks)	1.63 (24 months)	Direct observation of usage

Walker et al[61].	UK, London	2.3 (4 weeks)	2.6 (4 weeks)	-	Direct observation of usage

Broughton[62]	UK, London	-	-	2.6 (36 months)	Direct observation of usage

Constant et al[63].	France, nationwide	4.2 (24 months)	2.2 (24 months)	-	Self report of usage

Foss et al[64].	US, North Carolina	11.0 (4 to 8 weeks)	11.8 (20 weeks)	-	Direct observation of usage

Mccartt et al. [42,65]	US, New York City	2.3 (24 weeks)	1.1 (16 weeks)	2.1 (12 months)	Direct observation of usage

Mccartt et al[38,44].	US, Washington DC	6.1 (12 weeks)	3.5 (12 weeks)	4.0 (12 months)	Direct observation of usage

Rajalin et al[66].	Finland, nationwide	55.8 (no data)	15.2 (no data)	20.0 (no data)	Self report of usage

Key: * data on under 18 year olds only

**Table 4:** Impact of legislation on compliance or injuries/fatalities reported by included studies on child restraints usage in vehicles

		Data for compliance		
		
Study	Location	Pre legislation % (sampling interval before legislation)	Post legislation % (sampling interval after legislation)	Method used to obtain data
Collarile et al[41].	Italy, North East	74.7 (12 months)	92.5 (12 months)	Self report of usage

Murrin et al[67].	USA, California	5.6 (no data)	11 (no data)	Direct inspection of usage
		
		**Data for injuries/fatalities**		

**Study**	**Location**	**Pre legislation (data span used)**	**Post legislation (data span used)**	**Method used to obtain data**

Rock et al[68].	USA, Illinois	301 (48 months)	293 (48 months)	Database (Illinois Department of Transportation)

Margolis et al[69].	USA, North Carolina	2.19% (66 months)	1.82% (104 months)	Database (North Carolina Collision Reports)

Desapriya et al[5].	Japan, nationwide	No change reported (24 months)	No change reported (24 months)	Database (Traffic Bureau of National Police Agency and Institute for Traffic Accident Research and Data Analysis)

In term of the 'threat' of insufficient enforcement resources (e.g. policing time and/or commitment) our included studies clearly indicated that where local police were willing to devote resources to enforcement, then sustained compliance is achievable[38,42-44].

The inferences we can make thus indicates that a ban on smoking in vehicles carrying children is not 'unenforceable' (as often claimed), but when the appropriate strategies are deployed in a sustained manner, especially in the context of a population that values protecting children, then it is not only enforceable but may even be 'self-enforcing'.

## Discussion

Our realist synthesis has indicated that it is possible to develop test and refine a coherent and transferable framework that might guide the decision making process. Our initial framework was able to make some inroads into understanding and inferring what would happen if our 'test' case study (banning smoking in vehicles carrying children) was to be considered. We hope that we have demonstrated that the value of such a framework is to bring a structured way of thinking about threats to legislation, deliberately encourage policy and decision makers to seek out any assumptions they may have, to consider underlying theories and the acceptability of any piece of legislation.

We were unable to find any comparable attempt at providing an evidence-based-policy framework such as ours. However, we acknowledge that some sections of our framework may be found in sources we have not uncovered and also as tacit knowledge within the heads of seasoned practitioners (e.g. advocates or legislators). We do however hope that our attempts to develop and test it on our one 'case study' will make a primordial tool that will be useful to policy and decisions makers less well versed in the arena of public health legislation.

### Limitations

In all realist syntheses there will necessarily be judgement involved as to the inferences that can be made from data found within included studies. As new evidence emerges, we hope that our framework will form a starting point from which others may wish to build and develop. To further enable this process, we have undertaken the review in as transparent a manner as possible so that others may see how we have arrived at our inferences and theory of threats. We are unlikely to have uncovered each and every relevant paper. However, this is not the goal of the search process in realist synthesis, where the acceptance is that searching is purposive [8] and that theory testing is feasible with a 'maximum variety sample'[14]. Our theory of threats was only tested on a single case study - namely a ban on smoking in vehicles carrying children. Whilst we anticipate that most threats might be transferable across other legislative areas, it is possible that if our framework were to be used within a different legislative arena, new threats might need to be added (such as for example 'broader political environment' or 'existing over-arching law and policies). As such we would suggest that our initial framework is best viewed as 'work in progress' and would benefit from additional testing and refinement through use and/or testing on other types of legislation. Our decision to test our framework against a proposed ban on smoking in vehicles carrying children introduces an additional reason for us to suggest further testing, as not all the threats we identified (in Figure 1) were relevant to our test case.

Within the included studies, there are a high number of self reported questionnaire studies that have contributed to our inferences in the public support and enforcement threats. Self reported questionnaire data is subject to a range of well recognised biases [45] and so whilst we have made a number of inferences from this data, we are aware that these may be less secure. In a similar vein, all of our included studies hailed from richer democratic nations many of which also had well developed tobacco control policies and legislation. The inferences we have made have been drawn from data derived from such contexts and so may act to limit transferability of our programme theory to (for example) low income countries with less developed tobacco control policies.

## Conclusion

Our realist synthesis has shown that it is possible to develop and test a coherent and transferable framework that may assist policy and decision makers to analyse and judge if a proposed public health legislative option is feasible and likely to succeed. Our framework is not definitive but does act as a starting point and with use and/or as further relevant evidence emerges, refinement is likely to be needed.

## List of abbreviations

NICE: National Institute for Health and Clinical Excellence; SHS: Second hand smoke

## Competing interests

The authors declare that they have no competing interests.

## Authors' contributions

GW, RP and LO all contributed substantially to conception design, acquisition of data, analysis and interpretation of data. The original draft was undertaken by GW and RP and LO revised it critically for important intellectual content. All authors have given final approval for the publication of this version.

## Authors' information

GW is a General Practitioner and Clinical Lecturer in Primary Care at UCL, United Kingdom. His main research interests are in the synthesis of evidence from complex interventions, with a specific focus on the use of realist synthesis and medical education.

RP is Professor of Social Research Methodology at the University of Leeds, United Kingdom. He is best known for his writing on evaluation methodology and evidence-based policy; publications include A Measure for Measures (1989), Realistic Evaluation (1997) and Evidence-Based Policy: A Realist Perspective (2006).

LO is a senior analyst at the National Institute for Health and Clinical Excellence, an independent organization responsible for providing guidance on promotion of good health and prevention and treatment of ill health in England.

## Pre-publication history

The pre-publication history for this paper can be accessed here:

http://www.biomedcentral.com/1471-2458/11/222/prepub

## Supplementary Material

Additional file 1 **Table S1 - Included studies' characteristics and areas of contribution to framework testing and refinement (by date order)**. This file contains a table of all the studies that contributed to our synthesis. The studies are listed in date order and in addition information is provided on; population studied; article type; aim of article; research method; and how each article contributed to theory testing. Click here for file
