# Supplementary material for: Policy guidance on threats to legislative interventions in public health: a realist synthesis
Source: BMC Public Health. 2011 Apr 10;11:222. doi: 10.1186/1471-2458-11-222 (PMC3079647; doi:10.1186/1471-2458-11-222)
Supplement: Additional file 1 — Table S1 - Included studies' characteristics and areas of contribution to framework testing and refinement (by date order). This file contains a table of all the studies that contributed to our synthesis. The studies are listed in date order and in addition information is provided on; population studied; article type; aim of article; research method; and how each article contributed to theory testing. [file 1471-2458-11-222-S1.DOC]

## Table 1 - Included studies’ characteristics and areas of contribution to framework testing and refinement (by date order)

|  |  |  |  |  | **Data provided by article to framework** | | | |
| --- | --- | --- | --- | --- | --- | --- | --- | --- |
|  |  |  |  |  | **testing by type of threat** | | | |
| **Study** | **Population** | **Article type** | **Aim of article** | **Research method** | **Problem misidentification** | **Lack of public support** | **Lobby group opposition** | **Enforcement issues** |
| Bowman et al. (1987)[1] | Australia | Primary research | Implement and compare the effectiveness of two interventions to increase the safety restraint use of preschool children. Coercion (letters, posters and threat of fines from police) vs. education of children to wear seatbelt | Quasi-experimental using direct observation of vehicles |  |  |  | x |
| Lavelle et al. (1992)[2] | USA | Primary research | Evaluate the Colorado Occupant Protection Project's (COPP) effectiveness for increasing police officers' rate of issuing citations for nonuse of a safety seat | Quasi experimental using database |  |  |  | x |
| US Environmental Protection Agency (1992)[3] | World-wide literature | Review report | Review the effects of SHS on lung cancer and other disorders | Commissioned review (from academics) | x |  |  |  |
| Decina et al. (1994)[4] | USA | Primary research | Evaluate the effect of locally funded education and enforcement programs aimed at increasing child-restraint use | Quasi-experimental using direct observation of vehicles and questionnaire |  | x |  | x |
| Bauman et al. (1995)[5] | Australia | Letter | N/A | N/A |  | x |  | x |
| Margolis et al. (1996)[6] | USA | Primary research | Assess the effect of the North Carolina law mandating that all front seat passengers use a safety belt on children 4 through 15 years of age. | Quasi experimental using database |  | x |  | x |
| Rock et al. (1996)[7] | USA | Primary research | Assess effect of the Illinois law on restraining children 0 to 4 on injury and death. | Quasi experimental using database |  |  |  | x |
| Norman et al. (1999)[8] | USA | Primary research | Determine if those who are most in need of household smoking restrictions are the ones who have them? | Questionnaire survey |  | x |  |  |
| Crone et al. (2000)[9] | Dutch | Primary research | Assess the prevalence of passive smoking in infancy. | Questionnaire survey | x | x |  |  |
| Chapman (2001)[10] | Australia | Discussion piece / review | Explain the need and role for advocacy in public health | N/A |  | x | x |  |
| Evans et al. (2001)[11] | N/A | Review | Systematic review on interventions to promote use of child safety seats, promote use of safety belts, and deter alcohol-impaired driving | Systematic review by US government agency |  |  |  | x |
| Kegler et al. (2002)[12] | USA | Primary research | Understand house-hold and car smoking restrictions in low-income, rural Native American and White households with young children | Questionnaire survey |  | x |  |  |
| Pope et al. (2002)[13] | USA | Primary research | Establish link between environmental PM2.5 and lung cancer | Cohort study | x |  |  |  |
| Walsh et al. (2002)[14] | Australia | Primary research | Assess community knowledge, attitudes and practices in relation to environmental tobacco smoke (ETS) especially in homes, private motor vehicles and licensed premises, and to document levels of support t for further government legislation | Questionnaire survey |  | x |  | x |
| Gehrman et al. (2003)[15] | N/A | Review | Summarizes empirical evidence for reducing residential ETS exposure in youth in 19 studies published between 1987 and 2002. | Systematic review |  | x |  |  |
| Johansson et al. (2003)[16] | Sweden | Letter | Highlight that SHS can produce very high levels of exposure | N/A |  | x |  |  |
| Mccartt et al. (2003)[17] | USA | Primary research | Measure any change in the use of handheld cell phones by New York State drivers before and after the implementation of the cell phone law | Quasi-experimental using direct observation of vehicles |  |  |  | x |
| Mcmillen et al (2003)[18] | USA | Primary research | 1) Describe the knowledge, attitudes, and practices of smokers and non-smokers regarding smoking bans and child ETS expo-sure in multiple public and private settings; 2) Report changes from 2000 –2001. | Questionnaire survey |  | x |  |  |
| Desapriya et al. (2004)[19] | Japan | Primary research | Measure the effectiveness, benefits and usage of safety seats for child passengers aged 1–5 years before and after legislation through child casualty data. | Quasi experimental using database |  |  |  | x |
| Feinson et al. (2004)[20] | USA | Primary research | Ascertain parental knowledge and attitudes to ETS | Questionnaire survey |  | x |  |  |
| Fong et al (2004)[21] | Canada, USA, UK, Australia. | Primary research | Investigate the experience of regret among smokers; we assess its prevalence and identify key predictors of regret among smokers in a large representative survey of smokers in four countries: | Questionnaire survey |  | x |  |  |
| Invernizzi et al. (2004)[22] | Italy | Primary research | Compare PM production from an ecodiesel exhaust and smoldering cigarettes. | Experimental air quality study - diesel emissions vs. SHS | x |  |  |  |
| Mccartt et al. (2004)[23] | USA | Primary research | Determine whether substantial short term declines in drivers’ use of handheld cell phones, after a state ban, were sustained one year later. | Quasi-experimental using direct observation of vehicles |  |  |  | x |
| Mcmillen et al (2004)[24] | USA | Primary research | 1) Identify rural-urban differences in smoking restrictions; 2) Determine disparities in protection from ETS could be accounted for by differences in knowledge of the health risks associated with ETS and support for smoking restrictions | Questionnaire survey |  | x |  |  |
| Murrin et al (2004)[25] | USA | Primary research | Compare pre-law and post-law use of booster seats as evidenced by data gathered during child restraint inspections, including booster use; misuse/error by booster type; age and weight of child; sibling seats inspected; and vehicle type | Quasi-experimental using direct observation of vehicles |  |  |  | x |
| Tong et al. (2004)[26] | N/A | Primary research | Examine how the tobacco industry has tried to influence the scientific evidence for the link between SIDS and SHS | Text analysis |  | x | x |  |
| Travers et al. (2004)[27] | USA | Primary research | Describes an assessment of changes in indoor air quality that occurred in 20 hospitality venues in western New York where smoking or indirect secondhand smoke exposure from an adjoining room was observed at baseline | Experimental air quality study | x |  |  |  |
| Carr et al. (2005)[28] | USA | Primary research | Determine tobacco habits and attitudes in a American Chinese population | Questionnaire survey |  | x |  |  |
| Freudenberg (2005)[29] | USA | Discussion piece / review | Put forward the case the corporations that damage our health should be tackled head on | N/A |  |  | x |  |
| Gillespie et al. (2005)[30] | New Zealand | Primary research | 1) Measure the level of exposure to SHS in New Zealand homes and cars; 2) Describe modifiable risk factors such as participants’ smoking behaviours in domestic settings; 3) Attitudes towards smoking restrictions, SHS, and smoking around others | Questionnaire survey |  | x |  | x |
| Johal et al. (2005)[31] | UK | Primary research | Assess the effects of mobile phone usage before and after legislation banning use in cars | Questionnaire survey |  |  |  | x |
| King et al. (2005)[32] | USA | Primary research | Investigates association between implementing a personal space smoking restriction for the home or automobile, and various socio-demographic, social, behavioral, and attitudinal variables. | Questionnaire study |  | x |  | x |
| Nathanson (2005)[33] | N/A | Primary research | Describe and explain the way opposition to the tobacco industry has change with time and in different countries | Analysis of opposition to tobacco |  | x | x |  |
| Pierce et al. (2005)[34] | USA | Primary research | Determine the knowledge level of Head Start providers, parents, and students about booster seats and directly observe booster seat use before and after a combined educational program and booster seat giveaway | Quasi-experimental using direct observation of vehicles and questionnaire survey |  |  |  | x |
| Rajalin et al. (2005)[35] | Finland | Primary research | Assess the impact of the law on phone usage and self-reported safety during the first few months and 16 months later to determine whether the initial level of compliance with the law had been sustained | Quasi-experimental using direct observation of vehicles and questionnaire survey |  |  |  | x |
| Seo et all. (2005)[36] | USA | Primary research | Investigate the attitudes toward the smoking ban in motor vehicles among a representative sample of the State of Indiana | Questionnaire survey | x | x |  | x |
| Staunton et al (2005)[37] | USA | Primary research | Document child passenger restraint use and seating position among children aged 0 to 12 years in Georgia and evaluate the efficacy of Georgia’s child restraint surveillance and legislation. | Single direct observation of vehicles and questionnaire survey |  |  |  | x |
| Thomson et al. (2005)[38] | New Zealand | Review | Review the evidence on knowledge and attitudes among the New Zealand public concerning secondhand smoke (SHS) and smoking in homes and cars | Narrative review |  |  | x |  |
| World Health Organization (2005a)[39] | N/A | Report | Provide international guidance as what counts as 'clean' air | WHO report | x |  |  |  |
| World Health Organization (2005b)[40] | N/A | Factsheet | Explain and justify WHO PM2.5 levels | N/A | x |  |  |  |
| Beck et al. (2006)[41] | USA | Primary research | Identify beliefs, driving personality dispositions, and behaviors that distinguish self-defined aggressive drivers from non-aggressive drivers. | Questionnaire survey |  |  |  | x |
| Broughton (2006)[42] | UK | Primary research | Identify rates of seat belt (front and rear) usage and mobile phone usage whilst driving in London drivers and compare this to the national rate | Single direct observation of vehicles |  |  |  | x |
| Edwards et al (2006)[43] | New Zealand | Primary research | Investigate levels of PM2.5 in cars in New Zealand under different conditions of smoking, ventilation and speed of travel. | Experimental air quality study | x |  |  |  |
| Edwards et al. (2006)[44] | UK | Primary research | Test hypothesis that pubs and bars from more deprived areas and non food-serving pubs have higher levels of particulate air pollution | Experimental air quality study | x |  |  |  |
| Fong et al. (2006)[45] | Ireland | Primary research | Evaluate the psychosocial and behavioural impact of the first ever national level comprehensive workplace smoke-free law, implemented in Ireland in March 2004. | Questionnaire survey |  | x |  |  |
| Gonzales et al. (2006)[46] | USA | Primary research | 1) Examines prevalence of home and automobile smoking bans; 2) Associations of mother's country of birth and smoking practices with home and automobile smoking bans and child ETS exposure | Questionnaire survey | x | x |  |  |
| Hussain et al. (2006)[47] | UK | Primary research | Assess mobile phone use in cars in the Birmingham area 2 years after implementation of legislation | Quasi-experimental using direct observation of vehicles |  |  |  | x |
| Ibrahim et al. (2006)[48] | USA | Review | Document the tobacco industry's litigation strategy to impede tobacco control media campaigns. | N/A |  | x | x |  |
| Jalleh et al. (2006)[49] | Australia | Primary research | N/A | Questionnaire survey |  | x |  | x |
| Martin et al. (2006)[50] | New Zealand | Primary research | To establish a reproducible method to estimate the point prevalence of smoking and second-hand smoke (SHS) exposure in cars, and to compare this prevalence between two areas of contrasting socioeconomic status. | Single direct observation of vehicles | x |  | x | x |
| Mccartt et al. (2006)[51] | USA | Primary research | To assess the effects of Washington, D.C. law prohibiting drivers’ use of hand-held cell phones on such use. | Quasi-experimental using direct observation of vehicles |  |  |  | x |
| Mccartt et al. (2006)[52] | N/A | Review | Identify trends in drivers’ phone use and to determine the state of knowledge about the safety consequences of such use. | Systematic review of 125 studies. |  |  |  | x |
| Mcevoy et al. (2006)[53] | Australia | Primary research | Explore the use and effects of using mobile phones while driving | Questionnaire survey |  |  |  | x |
| Rees et al. (2006)[54] | USA | Primary research | Simulate children’s exposure to secondhand smoke in a motor vehicle by measuring carbon dioxide and respirable suspended particles (RSPs) of less than 2.5 microns in diameter, under actual driving conditions | Experimental air quality study | x | x |  |  |
| Vardavas et al. (2006)[55] | Greece | Letter | Measurement of PM2.5 in cars | Experimental air quality study | x |  |  |  |
| Walker et al. (2006)[56] | UK | Primary research | Assess the level of compliance with the new law in the United Kingdom mandating penalties for using a hand held mobile phone while driving, to compare compliance with this law with the one on the use of seat belts, and to compare compliance with these laws between drivers of four wheel drive vehicles and drivers of normal cars. | Quasi-experimental using direct observation of vehicles |  |  |  | x |
| Beck et al. (2007)[57] | USA | Primary research | Identify risky driving behaviors and dispositions that distinguish drivers who use a cell phone while operating a motor vehicle from non-cell phone using drivers | Questionnaire survey |  |  |  | X |
| Mccartt et al. (2007)[51] | USA | Primary research | To determine whether the substantial short-term declines in drivers’ use of hand-held phones achieved in the District of Columbia (DC) were sustained 1 year after a ban. | Quasi-experimental using direct observation of vehicles |  |  |  | X |
| Nabi et al. (2007)[58] | France | Primary research | Test the hypothesis that behavioral predictors of serious road traffic crashes (RTC) are correlated with unfavorable attitudes towards traffic safety | Questionnaire survey |  |  |  | X |
| Phillips et al (2007)[59] | UK | Primary research | Explore the accounts of smokers and non-smokers (who live with smokers) of smoking in their homes and cars after the Scottish smoke-free legislation; to examine the reported impact of the legislation on smoking in the home; and to consider the implications for future initiatives aimed at reducing children’s exposure to secondhand smoke in the home. | Qualitative interviews |  | x |  |  |
| Sly et al. (2007)[60] | Australia | Primary research | Determine relationship between wheezing and SHS exposure in cars and the home | Questionnaire study (reported in a letter) | x | x |  |  |
| Taylor et al. (2007)[61] | Australia | Primary research | To evaluate change in handheld mobile telephone (mobile) use among motor vehicle drivers between 2002 and 2006. | Single direct observation of vehicles |  |  |  | x |
| US Surgeon General (2007)[62] | USA | Report | Excerpts from larger report on SHS and its consequences with a focus on children | US Government report | x | x |  |  |
| Wilson et al. (2007)[63] | New Zealand | Primary research | (i) Assess compliance with the new smokefree law in a range of hospitality settings 18 months after the law came into force; and (ii) to assess the impact of the new law by measuring air quality and making comparisons with air quality in outdoor smoking areas and with international data from hospitality set-tings. | Experimental air quality study | x |  |  |  |
| Belanger et al. (2008)[64] | Canada | Primary research | Determine if SHS exposure might lead to the development of nicotine dependence symptoms in the absence of tobacco use among young never-smokers | Questionnaire survey | x |  |  |  |
| Bryan-Jones et al. (2008)[65] | Australia | Primary research | Examine the recent history of incremental legislation to introduce smoke-free indoor environments in the most populous Australian state of New South Wales (NSW) | Qualitative interviews |  | x | x |  |
| Chapman et al. (2008)[66] | N/A | Review | Explain and describe denormalisation and its consequences with reference to tobacco control | Narrative analysis |  | x | x |  |
| Collarile et al. (2008)[67] | Italy | Primary research | Evaluate the changes in prevalence of child-restraint correct use, level of parental information and parental driving behaviours | Questionnaire survey |  | x |  | x |
| Dunn et al. (2008)[68] | Australia | Primary research | Assess knowledge and attitudes and self reported behaviours with regards to ETS in cars and homes | Questionnaire survey |  | x |  |  |
| Enomoto et al. (2008)[69] | N/A | Review | Review of the evidence that PM2.5 from tobacco smoke causes cancer | Review | x |  |  |  |
| Freeman et al. (2008)[70] | Australia | Review | Summarise the 12-year Australian history of advocacy for banning smoking in cars carrying children | Qualitative narrative review of newspaper articles |  | x | x | x |
| Gallus et al. (2008)[71] | Europe | Review | Review evidence of link between PM and lung cancer from European studies. | Review | x |  |  |  |
| Jarvie et al. (2008)[72] | Mainly US focus | Review | Explore the ethical dimensions of SHS exposure in children when the exposure occurs in private homes and cars | N/A |  | x |  |  |
| Kegler et al. (2008)[73] | USA | Primary research | Explore car smoking rules and how they were established and enforced | Qualitative interviews | x | x |  |  |
| Leatherdale et al. (2008)[74] | Canada | Primary research | (a) Characterise the frequency of youth being exposed to smoking in their homes and cars, (b) characterise the prevalence of different beliefs youth have about smoking around children in their homes and cars, and (c) examine if current exposure to smoking in the home or in cars is associated with those beliefs. | Questionnaire survey | x | x |  |  |
| Lee et al. (2008)[75] | USA | Primary research | Estimate the prevalence of self-reported secondhand smoke (SHS) exposures and its association with respiratory symptoms in a sample of young adults residing in a state with a partial clean indoor air law (Florida) | Questionnaire survey | x | x |  | x |
| Letourneau et al. (2008)[76] | USA | Primary research | Evaluate a 3 year programme to improve child safety in cars through the use of proper restraints in American Indian (AI) and Alaskan Native (AN communities) | Programme evaluation (multiple methods) |  |  |  | x |
| Matt et al (2008a)[77] | USA | Primary research | Examine (a) the proportion of used cars sold in the private market that may be polluted with tobacco smoke and (b) whether asking prices of smoker and nonsmoker cars differed for cars of otherwise equivalent value | Questionnaire survey | x | x |  |  |
| Matt et al. (2008b)[78] | USA | Primary research | Examine whether cars of smokers are contaminated with residual secondhand smoke and whether contamination levels are affected by smoking behavior and restrictions | Experimental air quality study | x | x |  |  |
| Mbulo (2008)[79] | USA | Primary research | 1) Examine the extent to which Nebraska public middle and high school students were exposed to secondhand smoke in 2002 and 2006; 2) Evaluate factors associated with this exposure; 3) Propose interventions. | Questionnaire survey | x | x |  |  |
| Moulton (2008)[80] | Canada | News article | News article explaining new law in Nova Scotia banning smoking in cars with under 18's | N/A |  | x |  | x |
| Ott et al. (2008)[81] | USA | Primary research | Measured the air change rates of stationary and moving vehicles under different ventilation conditions and window positions | Experimental air quality study | x | x |  |  |
| Thomson et al. (2008)[82] | New Zealand | Primary research | Report on New Zealanders' level of support for banning smoking in cars | Questionnaire survey (reported in a letter) |  | x |  | x |
| Walsh et al. (2008)[83] | Australia | Primary research | 1) Assess community attitudes towards smoking bans, tobacco availability, promotion and product regulation, tobacco industry donations to political parties, and government spending on tobacco control activities. 2) Compare public preferences on these issues with policies of the NSW and Commonwealth governments. | Questionnaire study | x | x |  |  |
| Anitsal et al (2009)[84] | USA | Primary research | Assess purchase, use, attitudes and characteristics of parents to booster seats | Questionnaire survey |  |  |  | x |
| Binns et al. (2009)[85] | USA | Primary research | Understand clinician influence on use of home and automobile smoking bans in homes of children living with a smoker. | Questionnaire survey |  | x |  |  |
| Bolte et al. (2009)[86] | Germany | Primary research | Disentangle the impact of several dimensions of socioeconomic position on the prevalence of children’s ETS exposure at three indoor environments and on the family’s home smoking policy | Questionnaire survey |  | x |  |  |
| Cheraghi (2009)[87] | N./A | Review | Outline the harm STS does to children | N/A | x |  |  |  |
| Constant et al. (2009)[88] | France | Primary research | ( 1) Identify which risky behaviors reported in 2001 were associated with traffic collisions resulting in injuries in the subsequent 5-year period, ( 2) assess how drivers' self-reports of these risky behaviors changed between 2001 and 2004, and ( 3) determine predictors of these changes. | Questionnaire survey |  |  |  | x |
| Desapriya et al. (2009)[89] | Canada | Letter | N/A | N/A |  | x |  | x |
| Evans et al. (2009)[90] | Canada | Primary research | Investigate associations between both home and vehicle ETS exposure with chronic bronchitis | Questionnaire survey | x | x |  |  |
| Foss et al. (2009)[91] | USA | Primary research | Examine the opinions of teenagers and parents regarding cell phone restrictions. | Quasi-experimental using direct observation of vehicles and questionnaire survey |  |  |  | x |
| Jones at al. (2009)[92] | USA | Primary research | Measure air nicotine concentrations in motor vehicles of smokers and non-smokers while they commute to and from work. | Experimental air quality study | x | x |  |  |
| Leatherdale et al. (2009)[93] | Canada | Primary research | Examine second-hand smoke (SHS) exposure and the beliefs youth have about being exposed to SHS in their home and in cars and explores changes in exposure and beliefs over time. | Questionnaire survey | x | x |  |  |
| Mantziou et al. (2009)[94] | Greece | Primary research | 1) Identify predictors of childhood SHS exposure due to parental smoking; 2) Investigate into the relationships between the parents’ characteristics and their children’s exposure to cigarette smoke inside the house or family car | Questionnaire survey | x | x |  |  |
| Marshall (2009)[95] | N/A | Discussion piece | N/A | N/A |  | x |  |  |
| Meyer et al. (2009)[96] | N/A | Review | Show effects of smoking bans on acute myocardial infarctions | Systematic review and Meta-analysis | x |  |  |  |
| Polichetti et al. (2009)[97] | N/A | Review | Review of the link between PM 10, 2.5 and 1 and cardiovascular disease (CVD) | Review | x |  |  |  |
| Sendzik et al (2009)[98] | Canada | Primary research | Quantify the levels of TSP exposure under controlled conditions using established methods, with the use of real-time PM2.5 monitoring devices in a variety of different cars under a broad range of ventilation and airflow conditions. | Experimental air quality study | x | x | x |  |
| Thomson et al. (2009)[99] | N/A | Review | Review of surveyed attitudes to legal restrictions around smoking in cars. | Narrative review of all global data of public attitudes to smoking restrictions in cars | x | x |  |  |
|  |  |  |  |  |  |  |  |  |
|  | Glossary of abbreviations: | | ETS - Environmental Tobacco Smoke |  |  |  |  |  |
|  |  |  | N/A - not applicable or not available |  |  |  |  |  |
|  |  |  | PM2.5 - particulate matter 2.5 micrometres in diameter |  |  |  |  |  |
|  |  |  | SHS - Second Hand Smoke |  |  |  |  |  |
|  |  |  | STS - Secondhand Tobacco Smoke |  |  |  |  |  |
|  |  |  | TSP - Tobacco Smoke Pollution |  |  |  |  |  |

Reference List

1. J Bowman, R Sanson-Fisher, G Webb: **Interventions in Preschools to Increase the Use of Safety Restraints by Preschool Children.** *Pediatrics* 1987, **79:** 103-109.

2. JM Lavelle, MF Hovell, MP West, DR Wahlgren: **Promoting law enforcement for child protection: a community analysis.** *Journal of Applied Behavior Analysis* 1992, **25:** 885-892.

3. Respiratory health effects of passive smoking: lung cancer and other disorders. 1992. Washington D.C., US Environmental Protection Agency.

Ref Type: Report

4. L Decina, M Temple, H Dorer: **Increasing child safety-seat use and proper use among toddlers: Evaluation of an enforcement program.** *Accident Analysis & Prevention* 1994, **26:** 667-673.

5. A Bauman, XC Chen, S Chapman, A Bauman, XC Chen, S Chapman: **Protecting children in cars from tobacco smoke.** *BMJ* 1995, **311:** 1164.

6. LH Margolis, J Bracken, JR Stewart: **Effects of North Carolina's mandatory safety belt law on children.** *Injury Prevention* 1996, **2:** 32-35.

7. S Rock: **Impact of the Illinois Child Passenger Protection Act: A retrospective look.** *Accident Analysis & Prevention* 1996, **28:** 487-492.

8. GJ Norman, KM Ribisl, B Howard-Pitney, KA Howard, GJ Norman, KM Ribisl, B Howard-Pitney, KA Howard: **Smoking bans in the home and car: Do those who really need them have them?** *Prev Med* 1999, **29:** 581-589.

9. MRH Crone: **Prevalence of passive smoking in infancy in the Netherlands.** *Patient Education and Counseling* 2000, **39:** Feb.

10. S Chapman: **Advocacy in public health: roles and challenges.** *Int J Epidemiol* 2001, **30:** 1226-1232.

11. CA Evans, Jr., JE Fielding, RC Brownson, MJ England, MT Fullilove, FA Guerra, AR Hinman, GJ Isham, GH Land, CS Mahan et al.: **Motor-vehicle occupant injury: strategies for increasing use of child safety seats, increasing use of safety belts, and reducing alcohol-impaired driving.** *Morbidity & Mortality Weekly Report* 2001, **Recommendations:** 1-14.

12. MC Kegler, LH Malcoe, MC Kegler, LH Malcoe: **Smoking restrictions in the home and car among rural Native American and white families with young children.** *Prev Med* 2002, **35:** 334-342.

13. CA Pope III, R Burnett, M Thun, E Calle, D Krewski, K Ito, G Thurston: **Lung Cancer, Cardiopulmonary Mortality, and Long-term Exposure to Fine Particulate Air Pollution.** *JAMA* 2002, **287:** 1132-1141.

14. RA Walsh, F Tzelepis, CL Paul, J McKenzie, RA Walsh, F Tzelepis, CL Paul, J McKenzie: **Environmental tobacco smoke in homes, motor vehicles and licensed premises: community attitudes and practices.** *Aust N Z J Public Health* 2002, **26:** 536-542.

15. C Gehrman, M Hovell: **Protecting children from environmental tobacco smoke (ETS) exposure: A critical review.** *Nicotine Tob Res* 2003, **5:** 289-301.

16. A Johansson, G Hermansson, J Ludvigsson: **When does exposure of children to tobacco smoke become child abuse?** *Lancet* 2003, **361:** 1828.

17. A McCartt, E Braver, L Greary: **Drivers' use of handheld cell phones before and after New York State's cell phone law.** *Preventive Medicine* 2003, **36:** 629-635.

18. RC McMillen, JP Winickoff, JD Klein, M Weitzman, RC McMillen, JP Winickoff, JD Klein, M Weitzman: **US adult attitudes and practices regarding smoking restrictions and child exposure to environmental tobacco smoke: changes in the social climate from 2000-2001.** *Pediatrics* 2003, **112:** e55-e60.

19. EB Desapriya, N Iwase, I Pike, M Brussoni, M Papsdorf: **Child motor vehicle occupant and pedestrian casualties before and after enactment of child restraint seats legislation in Japan.** *Injury Control & Safety Promotion* 2004, **11:** 225-230.

20. JG Feinson: **Knowledge and attitudes about smoking among children with lung and allergic disorders and their parents.** *Pediatric Asthma, Allergy and Immunology* 2004, **17:** 2004.

21. G Fong, D Hammond, F Laux, M Zanna, K Cummings, R Borland, H Ross: **The near-universal experience of regret among smokers in four countries: Findings from the International Tobacco Control Policy Evaluation Survey.** *Nicotine Tob Res* 2004, **6:** S341-S351.

22. G Invernizzi, A Ruprecht, R Mazza, E Rossetti, A Sasco, S Nardini, R Boffi: **Particulate matter from tobacco versus diesel car exhaust: an educational perspective.** *Tob Control* 2004, **13:** 219-221.

23. AT McCartt, LL Geary: **Longer term effects of New York State's law on drivers' handheld cell phone use.** *Injury Prevention* 2004, **10:** 11-15.

24. R McMillen, J Breen, AG Cosby, R McMillen, J Breen, AG Cosby: **Rural-urban differences in the social climate surrounding environmental tobacco smoke: a report from the 2002 Social Climate Survey of Tobacco Control.** *J Rural Health* 2004, **20:** 7-16.

25. P Murrin, L Gardina: **Impact of new booster seat law.** *Journal of Emergency Nursing* 2004, **30:** 411-412.

26. E Tong, L England, S Glantz: **Changing Conclusions on Secondhand Smoke in a Sudden Infant Death Syndrome Review Funded by the Tobacco Industry.** *Pediatrics* 2004, **115:** e356-e366.

27. M Travers, K Cummings, A Hyland, J Repace, T Pechacek, R Cacaballo, S Babb: **Indoor Air Quality in Hospitality Venues Before and After Implementation of a Clean Air Law.** *Oncology Times* 2004, **26:** 52-55.

28. K Carr, M Beers, T Kassebaum, MJr Chen. California Chinese American Tobacco Use Survey - 2004. 2005. Sacramento, CA, California Department of Health Services.

Ref Type: Report

29. N Freudenberg: **Public Health Advocacy to Change Corporate Practices: Implications for Health Education Practice and Research.** *Health Educ Behav* 2005, **32:** 298-319.

30. JM Gillespie: **Secondhand smoke in New Zealand homes and cars: Exposure, attitudes, and behaviours in 2004.** *N Z Med J* 2005, **118:** 06.

31. S Johal, F Napier, J Britt-Compton, T Marshall: **Mobile phones and driving.** *Journal of Public Health* 2005, **27:** 112-113.

32. G King, R Mallett, L Kozlowski, RB Bendel, S Nahata, G King, R Mallett, L Kozlowski, RB Bendel, S Nahata: **Personal space smoking restrictions among African Americans.** *Am J Prev Med* 2005, **28:** 33-40.

33. C Nathanson: **Collective Actors and Corporate Targets in Tobacco Control: A Cross-National Comparison.** *Health Education & Behavior* 2005, **32:** 337-354.

34. SE Pierce, MP Mundt, NM Peterson, ML Katcher: **Improving awareness and use of booster seats in Head Start families.** *WMJ* 2005, **104:** 46-51.

35. S Rajalin, H Summala, L Poysti, P Anteroinen, B Porter: **In-Car Cell Phone Use and Hazards Following Hands Free Legislation.** *Traffic Injury Prevention* 2005, **6:** 225-229.

36. DC Seo, DC Seo: **Correlates of attitudes toward a smoking ban in vehicles.** *J Public Health Manag Pract* 2005, **11:** 346-350.

37. C Staunton, S Davidson, S Kegler, L Dawson, K Powell, A Dellinger: **Critical gaps in child passenger safety practices, surveillance, and legislation: Georgia, 2001.** *Pediatrics* 2005, **115:** 372-379.

38. G Thomson, N Wilson, P Howden-Chapman: **Attitudes to, and knowledge of, secondhand smoke in New Zealand homes and cars.** *N Z Med J* 2005, **118:** 1213.

39. World Health Organization. WHO Air quality guidelines for particulate matter, ozone, nitrogen dioxide and sulfur dioxide: Summary of risk assessment. 2005. Geneva, World Health Organization.

Ref Type: Report

40. World Health Organization. Particulate matter air pollution: how it harms health. 2005. Bonn, Germany, World Health Organization Europe.

Ref Type: Report

41. K Beck, MQ Wang, M Mitchell: **Concerns, dispositions and behaviors of aggressive drivers: What do self-identified aggressive drivers believe about traffic safety?** *Journal of Safety Research* 2006, **37:** 159-165.

42. J Broughton: **Mobile phone and seat belt usage rates in London, March 2006.** *Transport Research Laboratory* 2006, **Aug:** 2006.

43. R Edwards, N Wilson, N Pierse: **Highly hazardous air quality associated with smoking in cars: New Zealand pilot study.** *The Journal of the New Zealand Medical Association* 2006, **119:** 2294.

44. R Edwards, C Hasselholdt, K Hargreaves, C Probery, R Holford, J Hart, M Van Tongeren, A Watson: **Levels of second hand smoke in pubs and bars by deprivation and food-serving status: a cross-sectional study from North West England.** *BMC Public Health* 2006, **6:** 42.

45. G Fong, A Hyland, R Borland, D Hammond, G Hastings, A McNeill, S Anderson, K Cummings, S Allwright, M Mulcahy et al.: **Reductions in tobacco smoke pollution and increases in support for smoke-free public places following the implementation of comprehensive smoke-free workplace legislation in the Republic of Ireland: findings from the ITC Ireland/UK Survey.** *Tob Control* 2006, **15:** 51-58.

46. M Gonzales, LH Malcoe, MC Kegler, J Espinoza, M Gonzales, LH Malcoe, MC Kegler, J Espinoza: **Prevalence and predictors of home and automobile smoking bans and child environmental tobacco smoke exposure: a cross-sectional study of U.S.- and Mexico-born Hispanic women with young children.** *BMC Public Health* 2006, **6:** 265.

47. K Hussain, J Al Shakarchi, A Mahmoudi, A Al Mawlawi, T Marshall: **Mobile phones and driving: a follow-up.** *Journal of Public Health* 2006, **28:** 395-396.

48. J Ibrahim, S Glantz: **Tobacco industry litigation strategies to oppose tobacco control media campaigns.** *Tob Control* 2006, **15:** 50-58.

49. G Jalleh, R Donovan, S Stewart, D Sullivan: **Is there public support for banning smoking in motor vehicles?** *Tob Control* 2006, **15:** 71.

50. J Martin, R George, K Andrews, P Barr, D Bicknell, E Insull, C Knox, J Liu, M Naqshband, K Romeril et al.: **Observed smoking in cars: a method and differences by socioeconomic area.** *Tob Control* 2006, **15:** 409-411.

51. AT McCartt, LA Hellinga, LL Geary: **Effects of Washington, D.C. law on drivers' hand-held cell phone use.** *Traffic Injury Prevention* 2006, **7:** 1-5.

52. AT McCartt, LA Hellinga, KA Bratiman: **Cell phones and driving: review of research.** *Traffic Injury Prevention* 2006, **7:** 89-106.

53. SP McEvoy, MR Stevenson, M Woodward: **Phone use and crashes while driving: A representative survey of drivers in two Australian states.[see comment].** *Medical Journal of Australia* 2006, **185:** 630-634.

54. VW Rees, GN Connolly, VW Rees, GN Connolly: **Measuring air quality to protect children from secondhand smoke in cars.** *Am J Prev Med* 2006, **31:** 363-368.

55. C Vardavas, M Linardakis, A Kafatos: **Environmental tobacco smoke exposure in motor vehicles: a preliminary study.** *Tob Control* 2006, **15:** 415.

56. L Walker, J Williams, K Jamrozik: **Unsafe driving behaviour and four wheel drive vehicles: Observational study.** *British Medical Journal 333(7558)()(pp 71-73), 2006 Date of Publication: 08 Jul 2006* 2006, **333:** 71-73.

57. KH Beck, F Yan, MQ Wang: **Cell phone users, reported crash risk, unsafe driving behaviors and dispositions: a survey of motorists in Maryland.** *Journal of Safety Research* 2007, **38:** 683-688.

58. H Nabi, SL Rachid, S Lafont, M Chiron, M Zins, E Lagarde: **Attitudes associated with behavioral predictors of serious road traffic crashes: results from the GAZEL cohort.** *Injury Prevention* 2007, **13:** 26-31.

59. R Phillips, A Amos, D Ritchie, S Cunningham-Burley, C Martin, R Phillips, A Amos, D Ritchie, S Cunningham-Burley, C Martin: **Smoking in the home after the smoke-free legislation in Scotland: qualitative study.[see comment].** *BMJ* 2007, **335:** 553.

60. PD Sly, M Deverell, MM Kusel, PG Holt, PD Sly, M Deverell, MM Kusel, PG Holt: **Exposure to environmental tobacco smoke in cars increases the risk of persistent wheeze in adolescents.** *Med J Aust* 2007, **186:** 322.

61. D Taylor, C MacBean, A Das, R Rosli: **Handheld mobile telephone use among Melbourne drivers.** *Medical Journal of Australia* 2007, **187:** 432-434.

62. US Surgeon General. Children and Secondhand Smoke Exposure: Excerpts from The Health Consequences of Involuntary Exposure to Tobacco Smoke. A Report of the Surgeon General. 2007. Atlanta, GA, US Department of Health and Human Services.

Ref Type: Report

63. N Wilson, R Edwards, A Maher, J Nathe, R Jalali: **National smokefree law in New Zealand improves air quality inside bars, pubs and restaurants.** *BMC Public Health* 2007, **7:** 85.

64. M Belanger, J O'Loughlin, CT Okoli, JJ McGrath, M Setia, L Guyon, A Gervais, M Belanger, J O'Loughlin, CTC Okoli et al.: **Nicotine dependence symptoms among young never-smokers exposed to secondhand tobacco smoke.** *Addict Behav* 2008, **33:** 1557-1563.

65. K Bryan-Jones, S Chapman: **Political dynamics promoting the incremental regulation of secondhand smoke: a case study of New South Wales, Australia.** *BMC Public Health* 2008, **6:** 192.

66. S Chapman, B Freeman: **Markers of the denormalisation of smoking and the tobacco industry.** *Tob Control* 2008, **17:** 25-31.

67. P Collarile, F Valent, S Di Bartolomeo, F Barbone: **Changes in child safety restraint use and parental driving behaviours in Italy.** *Acta Paediatrica* 2008, **97:** 1256-1260.

68. J Dunn, S Greenbank, M McDowell, C Mahoney, P Mazerolle, S Occhipinti, S Steginga, J Dunn, S Greenbank, M McDowell et al.: **Community knowledge, attitudes and behaviours about environmental tobacco smoke in homes and cars.** *HEALTH PROMOT J AUST* 2008, **19:** 113-117.

69. M Enomoto, WJ Tierney, K Nozaki: **Risk of human health by particulate matter as a source of air pollution--comparison with tobacco smoking. [Review] [109 refs].** *J Toxicol Sci* 2008, **33:** 251-267.

70. B Freeman, S Chapman, P Storey: **Banning smoking in cars carrying children: an analytical history of a public health advocacy campaign.** *Aust N Z J Public Health* 2008, **32:** 60-65.

71. S Gallus, E Negri, P Boffetta, JK McLaughlin, C Bosetti, C La Vecchia: **European studies on long-term exposure to ambient particulate matter and lung cancer.** *Eur J Cancer Prev* 2008, **17:** 191-194.

72. JA Jarvie, RE Malone: **Children's secondhand smoke exposure in private homes and cars: an ethical analysis.** *Am J Public Health* 2008, **98:** 2140-2145.

73. MC Kegler, C Escoffery, S Butler, MC Kegler, C Escoffery, S Butler: **A qualitative study on establishing and enforcing smoking rules in family cars.** *Nicotine Tob Res* 2008, **10:** 493-497.

74. ST Leatherdale, P Smith, R Ahmed, ST Leatherdale, P Smith, R Ahmed: **Youth exposure to smoking in the home and in cars: how often does it happen and what do youth think about it?** *Tob Control* 2008, **17:** 86-92.

75. DJ Lee, NA Dietz, KL Arheart, JD Wilkinson, JD Clark, III, AJ Caban-Martinez, DJ Lee, NA Dietz, KL Arheart, JD Wilkinson et al.: **Respiratory effects of secondhand smoke exposure among young adults residing in a "clean" indoor air state.** *J Community Health* 2008, **33:** 117-125.

76. RJ Letourneau, CE Crump, JM Bowling, DM Kuklinski, CW Allen: **Ride Safe: a child passenger safety program for American Indian/Alaska Native children.** *Maternal & Child Health Journal* 2008, **12:** Suppl-63.

77. G Matt, R Romero, D Ma, P Quintana, M Hovell, M Donohue, K Messer, S Salem, M Aquilar, J Boland et al.: **Tobacco use and asking prices of used cars: prevalence, costs, and new opportunities for changing smoking behavior.** *Tobacco Induced Diseases* 2008, **4:** 2.

78. G Matt, P Quintana, M Hovell, D Chatfield, D Ma, R Romero, A Uribe: **Residual tobacco smoke pollution in used cars for sale: Air, dust, and surfaces.** *Nicotine Tob Res* 2008, **10:** 1467-1475.

79. L Mbulo, L Mbulo: **Changes in exposure to secondhand smoke among youth in Nebraska, 2002-2006.** *Prev Chronic Dis* 2008, **5:** A84.

80. D Moulton, D Moulton: **Stop that smoking car!** *CMAJ* 2008, **178:** 19.

81. W Ott, N Klepers, P Switzer: **Air change rate of motor vehicles and in-vehicle pollutant concentrations from second-hand smoke.** *Journal of Exposure Science and Environmental Epidemiology* 2008, **18:** 312-325.

82. G Thomson, N Wilson, D Weerasekera, R Edwards, G Thomson, N Wilson, D Weerasekera, R Edwards: **Ninety-six percent of New Zealand smokers support smokefree cars containing preschool children.** *N Z Med J* 2008, **121:** 139-140.

83. RA Walsh, CL Paul, F Tzelepis, E Stojanovski, A Tang, RA Walsh, CL Paul, F Tzelepis, E Stojanovski, A Tang: **Is government action out-of-step with public opinion on tobacco control? Results of a New South Wales population survey.** *Aust N Z J Public Health* 2008, **32:** 482-488.

84. M Anitsal, I Anitsal, K Liska: **Determinants of Booster Seat Usage: What Makes Parents Commit to Using Booster Seats on a Regular Basis.** *Journal of Management and Marketing Research* 2009, **2**.

85. HJ Binns, J O'Neil, I Benuck, AJ Ariza, Pediatric Practice Research Group., HJ Binns, J O'Neil, I Benuck, AJ Ariza, Pediatric Practice Research Group.: *Patient Educ Couns* 2009, **74:** 272-276.

86. GF Bolte: **Socioeconomic determinants of children's environmental tobacco smoke exposure and family's home smoking policy.** *European Journal of Public Health* 2009, **19:** January.

87. MS Cheraghi: **Environmental tobacco smoke (ETS) and respiratory health in children.** *Eur J Pediatr* 2009, **168:** August.

88. A Constant, LR Salmi, S Lafont, M Chiron, E Lagarde: **Road casualties and changes in risky driving behavior in France between 2001 and 2004 among participants in the GAZEL cohort.** *American Journal of Public Health* 2009, **99:** 1247-1253.

89. E Desapriya, K Turcotte, S Subzwari, I Pike: **Smoking inside vehicles should be banned globally.** *Am J Public Health* 2009, **99:** 1158-1159.

90. J Evans, Y Chen, J Evans, Y Chen: **The association between home and vehicle environmental tobacco smoke (ETS) and chronic bronchitis in a Canadian population: the Canadian Community Health Survey, 2005.** *Inhal Toxicol* 2009, **21:** 244-249.

91. RD Foss, AH Goodwin, AT McCartt, LA Hellinga: **Short-term effects of a teenage driver cell phone restriction.** *Accident Analysis & Prevention* 2009, **41:** 419-424.

92. M Jones, A Navas-Acien, J Yuan, P Breysse: **Secondhand tobacco smoke concentrations in motor vehicles: a pilot study.** *Tob Control* 2009, **18:** 399-404.

93. ST Leatherdale, R Ahmed, ST Leatherdale, R Ahmed: **Second-hand smoke exposure in homes and in cars among Canadian youth: current prevalence, beliefs about exposure, and changes between 2004 and 2006.** *Cancer Causes Control* 2009, **20:** 855-865.

94. V Mantziou, CI Vardavas, E Kletsiou, KN Priftis, V Mantziou, CI Vardavas, E Kletsiou, KN Priftis: **Predictors of childhood exposure to parental secondhand smoke in the house and family car.** *Int J Environ Res Public Health* 2009, **6:** 433-444.

95. M Marshall: **Practice, politics, and possibilities.** *British Journal of General Practice* 2009, **59:** 605-612.

96. D Meyers, J Neuberger, J He: **Cardiovascular Effects of Bans on Smoking in Public Places: A Systematic review and Meta-Analysis.** *Journal of the American College of Cardiology* 2009, **54:** 1249-1255.

97. G Polichetti, S Cocco, A Spinali, V Trimarco, A Nunziata, A Nunziata: **Effects of particulate matter (PM(10), PM(2.5) and PM(1)) on the cardiovascular system. [Review] [132 refs].** *Toxicology* 2009, **261:** 1-8.

98. T Sendzik, GT Fong, MJ Travers, A Hyland, T Sendzik, GT Fong, MJ Travers, A Hyland: **An experimental investigation of tobacco smoke pollution in cars.** *Nicotine Tob Res* 2009, **11:** 627-634.

99. G Thomson, N Wilson: **Public attitudes to laws for smoke-free private vehicles: a brief review.** *Tob Control* 2009, **18:** 256-261.
